# Characterising a human endogenous retrovirus(HERV)-derived tumour-associated antigen: enriched RNA-Seq analysis of HERV-K(HML-2) in mantle cell lymphoma cell lines

**DOI:** 10.1186/s13100-020-0204-1

**Published:** 2020-02-07

**Authors:** Witold Tatkiewicz, James Dickie, Franchesca Bedford, Alexander Jones, Mark Atkin, Michele Kiernan, Emmanuel Atangana Maze, Bora Agit, Garry Farnham, Alexander Kanapin, Robert Belshaw

**Affiliations:** 1 Peninsula Medical School, Faculty of Health: Medicine, Dentistry and Human Sciences, University of Plymouth, Plymouth, UK; 2 School of Biomedical Sciences, Faculty of Health: Medicine, Dentistry and Human Sciences, University of Plymouth, Plymouth, UK; 3 Department of Oncology, University of Oxford, Oxford, UK; 4 Current address: Institute of Translational Biomedicine, Saint Petersburg State University, Saint Petersburg, Russia

**Keywords:** HERV-K(HML-2), HERV-K, Transposable element, Cancer immunotherapy, Leukemia, NGS, minION, Transcriptomics, RNA-Seq

## Abstract

**Background:**

The cell-surface attachment protein (Env) of the HERV-K(HML-2) lineage of endogenous retroviruses is a potentially attractive tumour-associated antigen for anti-cancer immunotherapy. The human genome contains around 100 integrated copies (called proviruses or loci) of the HERV-K(HML-2) virus and we argue that it is important for therapy development to know which and how many of these contribute to protein expression, and how this varies across tissues. We measured relative provirus expression in HERV-K(HML-2), using enriched RNA-Seq analysis with both short- and long-read sequencing, in three Mantle Cell Lymphoma cell lines (JVM2, Granta519 and REC1). We also confirmed expression of the Env protein in two of our cell lines using Western blotting, and analysed provirus expression data from all other relevant published studies.

**Results:**

Firstly, in both our and other reanalysed studies, approximately 10% of the transcripts mapping to HERV-K(HML-2) came from Env-encoding proviruses. Secondly, in one cell line the majority of the protein expression appears to come from one provirus (12q14.1). Thirdly, we find a strong tissue-specific pattern of provirus expression.

**Conclusions:**

A possible dependency of Env expression on a single provirus, combined with the earlier observation that this provirus is not present in all individuals and a general pattern of tissue-specific expression among proviruses, has serious implications for future HERV-K(HML-2)-targeted immunotherapy. Further research into HERV-K(HML-2) as a possible tumour-associated antigen in blood cancers requires a more targeted, proteome-based, screening protocol that will consider these polymorphisms within HERV-K(HML-2). We include a plan (and necessary alignments) for such work.

## Background

Human Endogenous Retroviruses (HERVs) are the descendants of retroviruses that have copied themselves into germline cells of our ancestors and thereby become inherited in a Mendelian fashion [1]. Subsequent proliferation of such germline infections over millions of years [2] has led to the remains of HERVs now making up 5% of our genome sequence (8% if we include some older entities such as MaLRs, which are related to HERVs) [3]. The proviruses (loci) of endogenous retroviruses, like those of their more familiar exogenous (horizontally transmitted) relatives, contain all the motifs required for transcription and translation of their several proteins. One lineage of HERVs originated ~ 35 million years ago and are called HERV-K(HML-2), simplified here to HML-2. Silencing of protein expression in HML-2 breaks down in several disease states [4] and copies of the viral attachment protein (Env) accumulate on the cell surface (where in typical retroviral fashion they would come to coat the viral particle as it budded through the lipid bilayer) [5]. This Env protein has attracted interest as a potential Tumour-Associated Antigen (TAA) because it is expressed in multiple tumour types but not healthy tissues [6] (except in the placenta [7]), and thus might offer a target for a broad-spectrum anti-cancer immunotherapy. For example, a mAB (monoclonal antibody) and a Chimeric Antigen Receptor (CAR) T-cell targeting this protein have shown promise in cell line and mouse model studies in breast cancer [8] and in melanoma [9]. We ignore the long debate over whether HML-2 actually contributes to tumour proliferation [10–14] and focus on it as a TAA for cell-killing immunotherapy.

To exploit fully the potential of the HML-2 Env protein as a TAA we need to characterise the underlying genetics, which are complex because HML-2 exists in the human genome as approximately 100 individual proviruses (we ignore here the more common relict forms of HERVs called solo LTRs, where LTR is Long Terminal Repeat). More specifically, it will be helpful to know which proviruses contribute to protein expression in different cancers, whether these proviruses are present in all human individuals, and how many proviruses in total contribute to protein expression. Knowing how many proviruses are contributing to protein expression is important because this number might affect how quickly resistance to an anti-HML-2 immunotherapy is acquired. For example, in acute lymphoblastic leukemias (ALL) we see relapses of CAR-T therapy directed against the surface CD19 protein that are caused, in some cases, by escape variants that lose surface expression of the CD19 epitope [15]. The mechanism behind this type of escape appears to be upregulation of an alternative splicing variant that leads to the epitope disappearing from the cell surface (CAR-T cells are MHC-independent) while retaining essential activity of the protein [16]. Importantly, the latter study found that the skipped exon often acquired premature stop codons or frameshift indels (insertion/deletions). We suggest that if CD19 had been a non-essential protein – like HML-2 Env – escape would have been achieved more quickly by such simple mutational inactivation. Env expression from multiple proviruses would delay this.

Although possibly not essential for T cell-based immunotherapy (see Discussion), antibody-based therapy requires complete proteins that can be trafficked to the cell surface and which contain the transmembrane domain near the C-terminus. Currently seven HML-2 proviruses with full-length *env* ORFs (Open Reading Frames) have been identified from bioinformatic analysis of the reference [17] and non-reference human genome sequences [18], and PCR screening of ethnically diverse DNA samples [19] (Table 1; Additional file 1). Of these seven known Env-encoding sequences, six have been shown by in vitro transduction experiments to be capable of producing proteins [23]. We consider that an eighth provirus (11q22.1) might contribute to Env expression. This provirus has a premature stop codon within the cytoplasmic tail of Env [23]. Deletion of the cytoplasmic tail in the analogous protein in some lentiviruses does not prevent transport of the protein to the cell surface [24, 25] but the effect in HML-2 (a betaretrovirus) is unknown. These eight proviruses are all Type 2 HML-2, which is the canonical form [20]: Type 1 HML-2 proviruses have a 292 nt deletion at the junction between the *pol* and *env* ORFs causing an in-frame fusion of the two ORFs [26]. Env is normally expressed via a spliced transcript and, although a Pol-Env fusion protein has been reported in primary leukemia cells and leukemia cell lines [27], this protein would lack the Env signal peptide [28] responsible for entry into the endoplasmic reticulum and subsequent transport to the cell surface.

**Table 1:** Details of HML-2 Env-encoding proviruses^a^

Provirus name	Other common names	Genome coordinates (orientation)	GenBank Accession	Percentage of population with provirus^b^	Provirus age (my)	Full-length ORFs
6q14.1	K109	chr6:78427019–36083(−)	AF164615	100	< 2	gag, env^c^
7p22.1a & b^d^	HML-2.HOM K108 L & R	chr7:4622057–40031(−)	AC072054	100	< 2	a = pol, env^c^ b = pol, env
8p23.1a	K115	chr8:7355397–64859(−)	AY037929	15	5^e^	pol, env^c,f^
11q22.1	K118	chr11:101565794–75259(+)	N/A	78	< 2	pol^g^
12q14.1	K119	chr12:58721242–30698(−)	AC074261	67	< 2	gag, env^c^
19p12b	K113	chr19:21841539^h^	AY037928	10–30	< 0.5	gag, pol, env^c^
19q11	ERVK-19	chr19:28128498–37361(−)	Y17833	100	< 5	gag, pol, env^c^
Xq21.33	N/A	chrX:93606603^h^	N/A	< 5	0.67–1.3	gag, pol, env

^a^Data including name from ref. [20]. (genome coordinates from GRCh37/hg19), unless otherwise indicated

^b^Data on proportion of individuals carrying full-length provirus taken from refs [18, 21, 22]; note, provirus 7p22.1 is polymorphic for the tandem duplication

^c^Protein expression shown by transfection [23] (12q14.1 & 19q11 identified as K74261 & K17833 respectively in that study)

^d^Tandem duplication

^e^LTR divergence suggests an age of 5-9my for 8p23.1a [20] but, because provirus is human-specific, the integration date must be at the lower boundary (human chimp divergence is ~5mya)

^f^The one nucleotide deletion in *gag* may be a sequencing error

^g^As mentioned in the main text, this provirus has a premature stop codon 38 amino acid positions before the normal terminus in *env*, which might not prevent expression at the cell surface

^h^Pre-integration site coordinates (Xq21.33 from ref. [18])

Of the above eight potentially Env-encoding proviruses, five are known to be insertionally polymorphic (Table 1), defined as a provirus that is present in some but not all individuals. This phenomenon and the recombination event that produces solo LTRs give us two types of polymorphism additional to the more familiar allelic polymorphism caused by substitutions and indels (insertion/deletions). It is only since systematic searches for insertionally polymorphic proviruses have been more recently carried out [18, 29, 30] that we are confident of having identified most of the proviruses likely to be encountered.

We present here the results of an enriched RNA-Seq analysis of several cancer cell lines (using both short- and long-read technologies) focusing on the relative expression of these eight proviruses. We chose to work with Mantle Cell Lymphomas (MCL) because several studies have reported elevated HML-2 expression in blood cancer patients and leukemia cell lines [12, 31]. We used three MCL cell lines expressing the potential TAA: JVM2, Granta519 (G519) and REC1. The first two lines are from early stage cases of MCL and the latter is from a late (indolent) stage. We also compare the general pattern of HML-2 provirus expression to other studies of cell-associated HML-2 expression and some whole transcriptome datasets from the same cell lines available from NCBI’s SRA (Short Read Archive). Studies of putative virion-associated HML-2 expression [32, 33] are excluded because such expression differs markedly from cell-associated expression in the source cells [34].

## Results

**Fig. 1:**
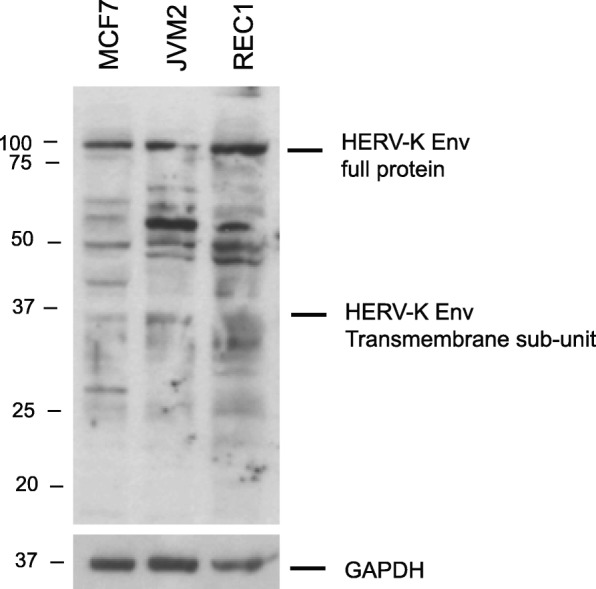
Western blot showing Env expression in JVM2 and REC1. MCF7 is present as a positive control. The uncleaved ~ 100 kDa full-length Env protein is clearly present in all cell lines. Other bands represent cleavage products, multiple glycosylation states and – at 55kDA – non-specific binding (see Methods)

We first confirmed Env protein expression in two of our cell lines using Western blotting with a widely used commercial mAB (Fig. 1). As a positive control we used the Breast Cancer cell line MCF7, which has previously been shown to express the protein (using a different mAB) [35].

**Fig. 2:**
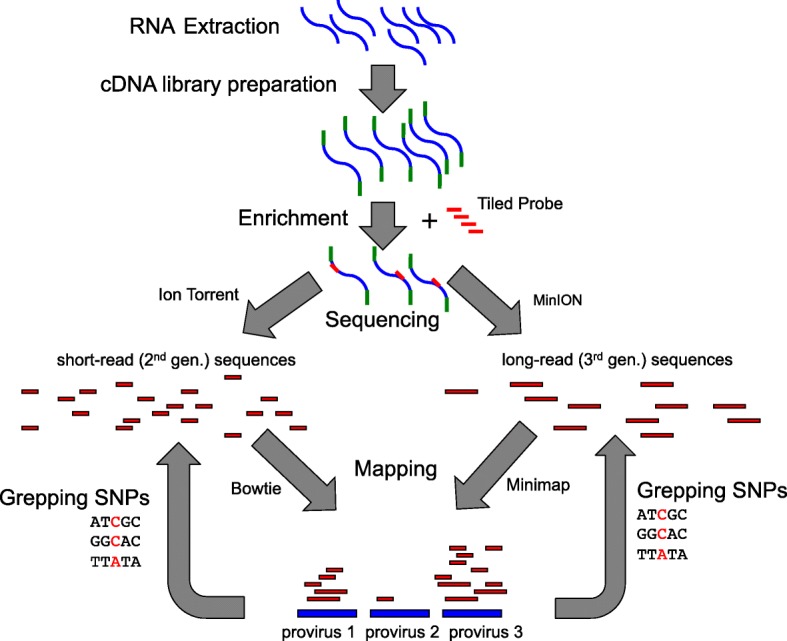
Illustrated summary of workflow in our study. See Methods for details

We then designed a probe DNA sequence using a consensus of the recently integrated HML-2 proviruses and used this to perform an enriched short-read RNA-Seq analyses of the three cell lines (Fig. 2). This analysis generated 2.7–3.1 million reads after Quality Control (3.1–3.3 million before), and 0.9–8.7% of these mapped to HML-2 (Table 2). The low percentage of assigned reads came from REC1 (the cell line from a slow growing [indolent] stage MCL). While > 80% of reads coming from target sequences are often reported for RNA-Seq enrichment using the same and similar technologies to ours [36], such values are typically for panels of genes that without enrichment would account for much more than our baseline of 0.003% of reads (the unenriched JVM2 control run in Table 2). For example, one array probe with 50 protein-coding loci resulted in 80.7% of captured reads coming from probed regions but this was only actually an ∼380-fold enrichment [37]. In the JVM2 cell line, our enrichment achieved an ~ 1000–3000-fold increase in the proportion of reads assigned to HML-2 compared to an unenriched control run, thereby giving us good coverage from small, economical sequencer runs. Enrichment also avoided a major artifact that we suspect was caused by the common presence of Alu insertions in old HML-2 proviruses (Additional file 2). We also carried out a single enriched long-read RNA-Seq analysis of the JVM2 cell line. This generated more than 200,000 reads of average length 2000nts but, as expected, with very high error rates (few reads with > 80% similarity to a provirus).

**Table 2:** Summary of sequencing results for the MCL cell lines^a^

Cell line	JVM2					G519	REC1
Sequencing method	Ion Torrent (short-read)				MinION (long-read)	Ion Torrent (short-read)	
Enrichment	No	Yes Growth 1	Yes Growth 2	Yes Growth 3	Yes	Yes	Yes
Total reads after QC	3,255,142	2,749,743	2,672,868	3,508,762	218,872	2,839,730	3,073,933
Total reads assigned to HML-2 (%)	113^b^ (0.003%)	155,700 (5.6%)	232,687 (8.7%)	101,495 (2.9%)	14,147 (6.9%)	113,807 (4.0%)	28,014 (0.91%)
Percentage reads from Env-encoding proviruses^c^	N/A	5.3%	15.9%	12.8%	20.8%	17.2%	2.3%

^a^Raw Ion Torrent run reports are shown in Additional file 3

^b^After excluding the probably artifactual 52 hits to provirus 9q34.3 (see Additional file 2)

^c^Percentage of reads mapping to Env-encoding proviruses was calculated after normalisation via conversion to RPKM values (Reads Per Kilobase of transcript per Million mapped reads)

**Fig. 3:**
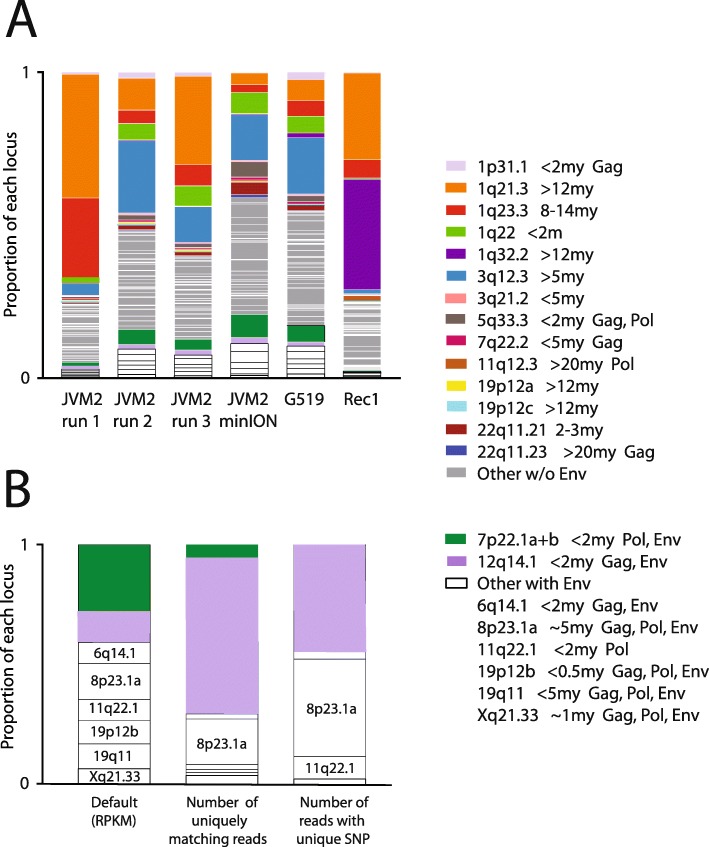
Relative expression of HML-2 proviruses in our study. **a** All experiments with default mapping. **b** Default mapping in JVM2 compared to counting only unique mappings and the results of a search for unique SNPs (data in Table 3; mean number of SNP hits calculated). Colours match those in Fig. 4. Env-encoding proviruses listed in same order in each bar. Provirus age and full-length ORFs indicated [20] (provirus 11q22.1 has a premature stop codon near its Env C-terminus). Genomic coordinates in Table 1 or as follows: 1p31.1 = chr1:75842771–9143; 1q21.3 = chr1:150605284–8361; 1q23.3 = chr1:160660575–9806; 1q22 = chr1:155596457–605636; 1q32.2 = chr1:207808457–12636; 3q12.3 = chr3:101410737–9859; 3q21.2 = chr3:125609302–18416; 5q33.3 = chr5:156084717–93896; 7q22.2 = chr7:104388369–93266; 11q12.3 = chr11:62135963–50563; 19p12a = chr19:20387400–97512; 19p12c = chr19:22757824–64561; 22q11.21 = chr22:18926187–35307; 22q11.23 = chr22:23879930–88810

To test for experimental bias we ran three short-read analyses of the JVM2 cell-line (Fig. 3a). These were all on independent growths of the cell line, and the only protocol difference in the short-read sequencing was a shortening of the RNA digestion step with JVM2 growth1 – leading to a 21% increase in median read length (Additional file 3). Although there were differences in expression of some proviruses, the ranked relative expressions of proviruses were very similar – even the least similar JVM2 experiments (1 and 2) were highly correlated (Spearman Rank Correlation Coefficient = 0.87).

### Env-encoding proviruses make up 10% of total HML-2 transcription

The eight Env-encoding proviruses made up 2% of the total HML-2 transcripts in REC1 and between 5 and 21% of the total transcripts in JVM2 and G519 (normalised using RPKM values – Reads Per Kilobase of transcript per Million mapped reads, Table 2). They also accounted for 13–14% of the (normalised) HML-2 reads from Illumina whole transcriptome RNA-Seq datasets of JVM2 and G519 cell lines downloaded from the SRA (Additional file 2), although the total number of reads mapping to HML-2 in each was much lower (only 1500-8000) than in our enrichment.

**Fig. 4:**
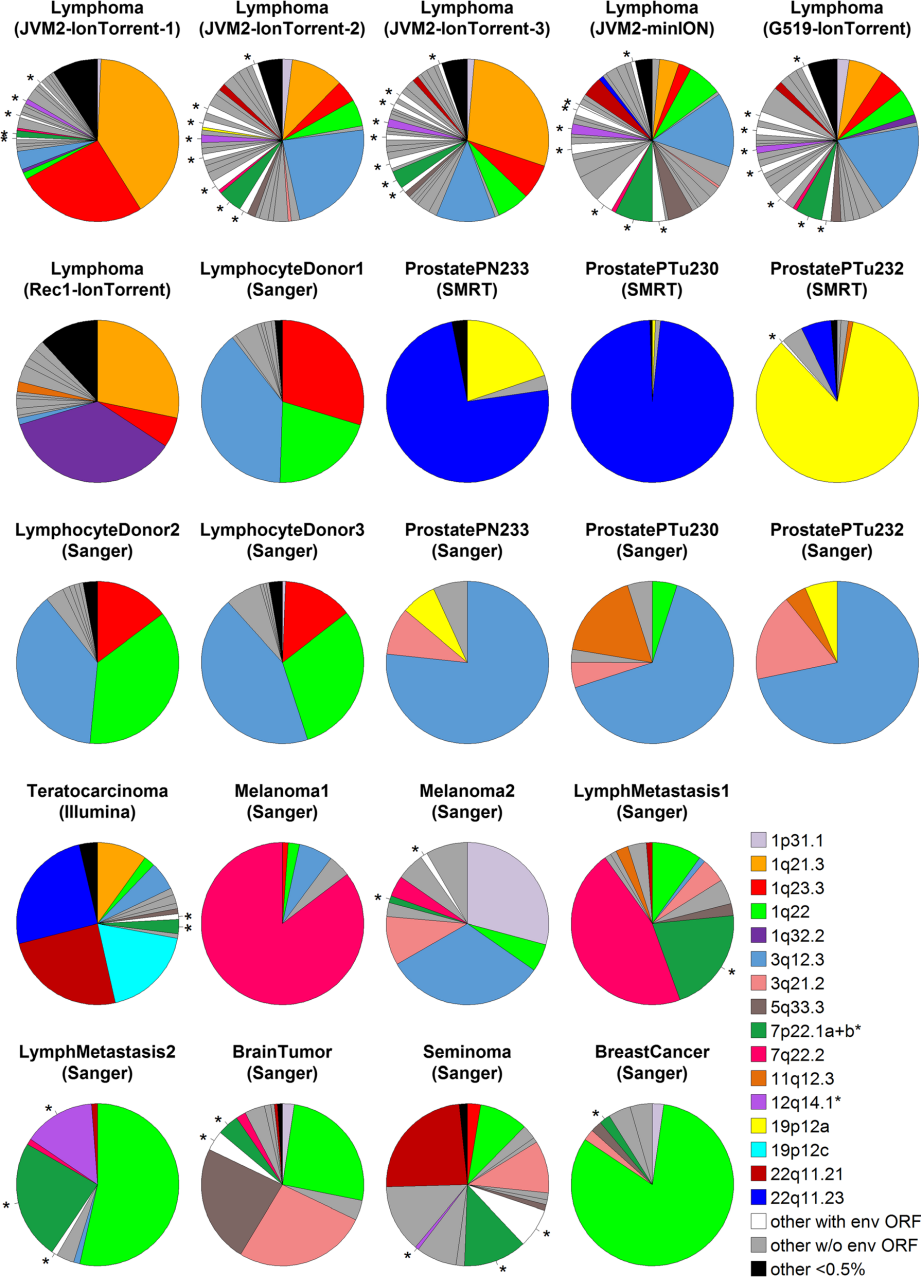
Relative expression of HML-2 proviruses in our and other studies. Relative expression of proviruses is shown as thickness of the pie slice. Env-encoding proviruses are indicated with an asterisk. Our three Mantle Cell Lymphoma cell lines – JVM2(Ion Torrent1–3 and minION), G519 and REC1 – are compared to published data from healthy donor lymphocytes, and other cancer cell lines and tissues (see text and Additional file 6 for details). The sequencing method is shown in parenthesis after the name. Results for Sanger and SMRT (Single Molecule Real Time) sequencing of three prostate biopsies are shown (one above the other) but note that the absence of provirus 22q11.23 from the Sanger sequencing is an artifact of the RT-PCR primers used (which incidentally were the same as those used in the melanoma and other cancers analysed by Sanger sequencing; note, PN233 is benign, the other two are cancerous). Results from two other lymphocyte donors not shown are very similar to the three shown here. The tandem duplication 7p22.1a + b (which have identical *env* sequences) are treated as one provirus in most studies so their expression values are combined here. Raw data available in Additional file 11

We find high relative expression across cancers of some Env-encoding proviruses (Fig. 4). Provirus 12q14.1 is highly expressed in a single lymph metastasis and the tandemly repeated provirus 7p22.1a + b has high relative expression in both lymph metastases plus a seminoma. The spliced Env-encoding transcript of 7p22.1a + b (identical in the two proviruses) was also found by RT-PCR and Sanger sequencing within the DU145 prostrate cancer cell line [38]. On average, Env-encoding proviruses account for 9% of the HML-2 transcription across the other published studies of expression in cancer shown in Fig. 4.

### Most potential Env expression in JVM2 comes from a single provirus

Closer inspection of our data from JVM2 reveals the difficulty of measuring the relative expression of very similar proviruses by both short and long reads. In Fig. 3b we compare the number of short reads mapped to Env-encoding proviruses by three methods: (i) default mapping, (ii) counting only reads that map uniquely (and reliably) to each provirus, and (iii) counting only reads that contain SNPs unique to one provirus (Table 3). The latter two methods show a majority of the reads coming from a single provirus (12q14.1). This finding is supported by an examination of the long reads that map reliably or which contain unique SNPs (Table 3), although the numbers here are low. The reason for this difference is that our eight Env-encoding proviruses differ from each other by only 1.5% on average at the nucleotide level. Many short reads therefore map equally well to multiple proviruses (illustrated in Additional file 4), and are randomly allocated to these by the default settings in the Bowtie2 program used in our analysis. Such reads contrast with those that map to a single provirus best (= uniquely mapping reads). No long reads map equally well to more than one provirus, but their high error rate leads to the same effect. This artefact can be removed by excluding long reads with low mapping quality (although this leaves us with only a few mapped reads). The more even distribution of reads among the Env-encoding proviruses shown in Figs. 3a and 4 therefore reflects random multi-mapping among these proviruses. Despite this mapping problem, we are confident that Env-encoding proviruses represent 10% of transcription because these proviruses are more similar to each other than they are to proviruses lacking full-length *env* ORFs (Additional file 5). Reads that are miss-mapped because of identity with multiple proviruses will therefore tend to be mapped to other Env-encoding proviruses.

**Table 3:** Determining which of the Env-encoding proviruses are likely to contribute to protein expression in the JVM2 cell line

Provirus	Number of uniquely mapping short reads (long reads in parentheses)^a^	Unique *env* SNP alleles^b^	Number of short reads with unique SNP allele (long reads in parentheses)^c^
6q14.1	31 (0)	T(655)C	0 (0)
		G(799)A	15 (0)
		G(806)A	9 (0)
7p22.1a + b^d^	83 (0)	C(51)T	2 (0)
		T(371)C	0 (0)
		A(1116)G	0 (0)
8p23.1a	280 (1)	C(293)T	0 (0)^e^
		G(958)A	365 (3)
		G(1707)C	47 (0)
		C(1983)T	1 (10)
11q22.1	33 (0)	C(537)	3 (0)^f^
		G(1804)A	0 (2)^f^
		G(2005)A	68 (3)^f^
12q14.1	962 (9)	G(96)A	275 (14)
		T(465)C	12 (0)
		C(586)A	89 (0)^g^
		C(1484)T	75 (0)
19p12b	17 (1)	C(421)T	0 (0)^h^
		C(970)G	0 (0)
		C(1885)A	0 (1)^i^
		A(1996)C	0 (0)
19q11	19 (1)	A(657)T	0 (1)
		T(1355)C	0 (1)
		T(1416)A	0 (0)
		T(1416)A	0 (0)^f^
Xq21.33	52 (0)	C(52)A	0 (0)
		G(827)A	16 (9)
		G(1219)A	0 (1)

^a^All multi-mapping short reads excluded. All long reads have a mapping quality score of at least 20 (equivalent to a mapping error of *p* = 0.01)

^b^Env SNPs with allele that is found only in a single provirus. Positions relate to the Env alignment available in Additional file 9 with ancestral state inferred by commonality. In a few instances there is a second SNP within the 31 nt sequence and it is the combination that is unique

^c^Average number of short-read matches to a 31 nt sequence spanning the SNP that are unique to the provirus, with corresponding result from the single minION run (17 nt match) in parenthesis

^d^Because these proviruses are almost identical (resulting from a recent tandem duplication), and hence each would have few uniquely mapping reads, we repeated the analysis with provirus 7p22.1a deleted

^e^SNP allele also present in unexpressed provirus Xq11.1

^f^SNP allele also present in several other proviruses

^g^SNP allele also in provirus 5p12, which has only 5 unique short-read hits

^h^SNP allele also in unexpressed provirus 1q24.1

^i^SNP allele also in the expressed provirus 6p21.1

Removing the multi-mapping artefact reveals an apparent absence of expression in several Env-encoding proviruses, which is consistent with what we know about their likely presence. For example, Xq21.33 is rare in the human population (allele frequency ~ 0.01 [18]) and only one of the three SNP alleles in its *env* sequence that are unique among the sequenced proviruses was found in more than one read (G827A). We similarly failed to find strong evidence for the uncommon 19p12b provirus (better known as K113).

**Fig. 5:**
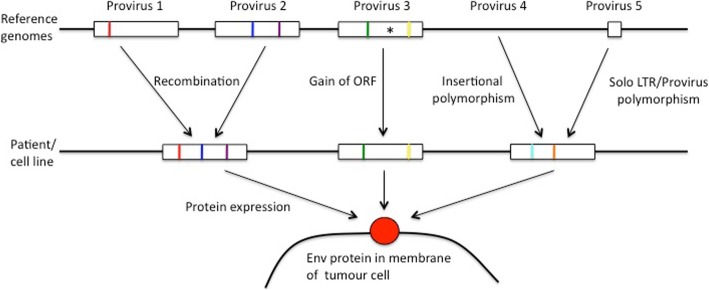
Problems in identifying proviruses from sequenced *env* transcripts or proteoforms. Hypothetical unique alleles in single nucleotide polymorphisms (SNPs) or single amino acid variants (SAAVs) are represented as coloured vertical bars (absence of the coloured bar denotes presence of the alternate variant) and premature stop codons represented as an asterisk. The figure shows possible difficulties that may arise in attempting to determine which proviruses gave rise to the Env protein in a patient or cell line. See Additional file 7 for further explanation of the mechanisms

Recovery of the G827A allele, but not the other two alleles thought to be unique to Xq21.33, indicates that G827A is actually present within another provirus in the individual from which our cell line is derived. The apparent uniqueness of G827A to Xq21.33 is thus an artifact of our limited sequencing of the proviruses in the human population. Typically, only one copy of each provirus within the human population has been published, so some nucleotide polymorphisms that are shared between different proviruses (in this case, two proviruses with the G827A allele) will not have been observed and may cause short reads to be miss-mapped if one provirus is absent. We found 59 long reads with matches to two *env* SNP alleles that were putatively unique to different proviruses. These reads probably represent previously unseen shared polymorphisms and could be generated by recombination between different HML-2 proviruses, for which there is evidence [39]. In Fig. 5 we illustrate the problems caused by the different types of polymorphism in HML-2 described in Background.

### Provirus expression is tissue-specific

Four proviruses lacking full-length *env* ORFs dominate expression in our MCL cell lines (accounting for 36–71% of reads): 1q21.3, 1q22, 1q23.3 and 3q12.3 (Figs. 3a and 4). Provirus 1q21.3 is missing most of *env*, 3q12.3 has multiple premature stop codons, and both 1q22 and 1q23.3 are Type 1 HML-2 s [20]. The similarity between JVM2 and G519 cell lines is also found in whole transcriptome RNA-Seq datasets downloaded from the Short Read Archive at https://www.ncbi.nlm.nih.gov/sra (Additional file 2), although these are based on far fewer matching reads than our analyses, and might reflect them both being derived from early stage tumours.

The unique high expression of 1q32.2 in REC1 is puzzling and discussed in Additional file 6. Three of our four more highly expressed proviruses were also found to dominate RNA expression in an earlier study of healthy lymphocytes [40] (Fig. 4), and the absence of the fourth (1q21.3; orange in Fig. 4) is probably an artifact caused by this provirus having a deletion at the position of the qPCR primers used in that study. The same study reported Env protein expression from transfected *env* sequences of both 1q22 and 1q23.3 using Western blotting (with the same commercial monoclonal antibody that we used here). This construction is in effect recreating the latter part of the Pol-Env fusion protein described in Background.

In contrast to the similarity between the expression pattern of HML-2 proviruses in our cell lines and in healthy lymphocytes, the pattern differed markedly from that reported by studies of other cancers (Fig. 4; Additional file 6). However, except for whole transcriptome (unenriched) RNA-Seq analysis of a teratocarcinoma cell line (Tera1) [34], these other studies – benign and cancerous prostate biopsies [41] and melanoma cell lines plus a range of cancer biopsies [42] – are based on an initial RT-PCR of a small HML-2 region. The overall pattern that emerges is of strong tissue- and cancer-specific transcription patterns (remarked on by previous authors [43]), with expression dominated by one to several proviruses, e.g. the Gag protein of 22q11.23 is a potential biomarker for prostate cancer [44] and a potentially oncogenic gene fusion between this provirus and (downstream) the ETV1 (ETS variant 1) transcription factor has been reported [45]. Another study using an initial RT-PCR to measure HML-2 expression in the brain of patients with a specific neurological disease [46] also showed a novel pattern: the single most highly expressed provirus was 7q34, which does not feature highly in our or the other studies.

## Discussion

In our JVM2 cell line, we suspect that much of the protein expression derives from the provirus 12q14.1, which is absent from a significant minority of the population (perhaps as high as one-third). This might explain why Schmitt et al. [42] found RNA expression of 12q14.1 in only one of five lymph node metastasis and melanoma samples. Similarly, Philippe et al. [47] found in a range of human cell lines that expression of L1s, another type of transposable element with thousands of loci in the human genome, was dominated by a very small number of loci, several of which are insertionally polymorphic. Future immunotherapy directed against HML-2 might therefore require an initial screen [48] to detect those patients in which a key provirus is absent or represented by a solo LTR.

We also need to investigate the mechanism of upregulation in key proviruses given the overall tissue-specific expression pattern. Many transcription factors are known, or are inferred, to bind to the HML-2 5′ LTR [49], e.g. expression of the 22q11.23 (H22q) provirus – the potential prostate cancer biomarker mentioned above – has been shown to be regulated by androgens via its androgen receptor binding site [41]. Epigenetic factors such as DNA methylation are also known to affect HML-2 expression [50]. LTR-driven expression of provirus 3q12.3 in human mammary epithelial cells was confirmed in vitro by a luciferase assay [51], but the same study showed 1q21.3 to be read-through transcribed (being situated downstream of another repetitive element). Other HML-2 proviruses were shown in that study to be expressed due to being within introns. Provirus 1q22, which is moderately expressed in our cell lines and highly expressed in the healthy lymphocyte study, was shown to be within a long non-coding RNA (lncRNA) known to be highly expressed in breast cancer [51]. We do not find strong evidence for the expression of the Env-encoding provirus 19q11 in the JVM2 cell line. This provirus appears to be fixed in the human population and it might not be expressed because it lacks all of its upstream promoter sites (the 5′ LTR is missing except for the last 23nts).

Although their amino acid sequences will be very similar, knowledge of expressing proviruses will help mAB design, e.g. the FEASK epitope identified in our mAB by Kämmerer et al. [7] is interrupted by a E to K mutation in the Env-encoding provirus 11q22.1, and the epitope of a mAB used in another study [27] has multiple amino acid polymorphisms in our alignment of Env-encoding proviruses.

Regarding future work, a large screening to measure Env protein expression in the blood/lymph tissue of cancer patients and healthy controls is now required. Ultimately, determining which proviruses contribute to protein expression requires proteomic analysis in which the constituent proteoforms would be identified and their amino acid sequence matched to the nucleotide sequence of the transcribing proviruses. We give guidance on how to approach this in Additional file 7. Bioinformatic investigation of the expanding number of whole genome sequences would also allow us to quantify the several issues with HML-2 polymorphism raised in our study.

Finally, in addition to a possible antibody-based therapy targeting Env, HML-2 might serve as a TAA via MHC-I antigen presentation in therapies directed at modifying T-cell responses to cancer, e.g. in a peptide vaccine. Cell-killing by cytotoxic T lymphocytes (CTLs) targeting HML-2 has been demonstrated in ex vivo tumour cells [52], and – from another line of medical research into HML-2 – in HIV-infected cells [53]. It would be useful to know the extent to which HML-2 proviruses truncated by premature stop codons (including those generated by frameshifting indels) are presented. One study found that a truncated Env from an old provirus belonging to a different (though related) ERV lineage, HERV-K(HML-6), was responsible for a T-cell response in a melanoma patient [54].

## Conclusions

Using a successful enrichment procedure, we found 10% of reads mapping to HML-2 were from Env-encoding proviruses. However, in one cell line we found that most of the protein expression appears to come from a single provirus, which is not present in all individuals. We believe that this insertional polymorphism, combined with a general tissue-specific pattern of expression, could have important therapy implications and that a proteomic analysis producing long amino acid reads is now required to definitively characterise this potentially wide-spectrum TAA.

## Methods

### Overview

We extracted and purified total cellular RNA from cell lines purchased from DSMZ (Deutsche Sammlung von Mikroorganismen und Zellkulturen GmbH), Braunschweig, Germany. Following this, cDNA library preparation and SureSelect targeted-sequencing were performed on Ion Torrent PGM (short-read) and minION (long-read) sequencers following standard protocols. Enrichment was done using a custom SureSelect RNA Target Enrichment kit with a consensus of the internal proviral regions (i.e. lacking the flanking LTRs) of the 20 HML-2 proviruses that had integrated within the last 5 million years (these include all the known Env-encoding proviruses; see Additional file 8). Designing the probe to the internal, protein-coding region, allowed us to avoid dilution of coverage caused by sequencing transcripts from the solo LTRs, which are not protein-coding but which are much more common than full-length proviruses and contain the motifs necessary to initiate transcription. To measure method consistency, we performed three sequencing runs with the Ion Torrent on the JVM2 cell line (each on a separate cell expansion).

Resulting single-end reads were then mapped to known sequences of the individual HML-2 proviruses. Subramanian et al. [20] gives details of 91 proviruses, which includes four proviruses not present within the reference human genome sequence [10p12.1, 12q13.2, 19p12b (=K113), and U219 (=K105)]. We added the sequences of four proviruses found subsequently [18]. Reads were mapped to these 95 proviruses in a ‘faux’ genome, consisting only of concatenated HML-2 sequences as in Bhardwaj et al. [34], using Bowtie2 [55] with the mappings counted using Cufflinks [56] (both run with default settings). An important aspect of the bioinformatic analysis is the need to retain multi-mapping reads. These are reads whose ‘best’ match is to more than one provirus, and Cufflinks by default allocates such multi-mapping reads randomly to potential targets. The Env-encoding proviruses have all integrated into our genome within the last few million years and hence have not had sufficient time to diverge from each other compared to the older, more degraded proviruses. Average pair-wise amino acid divergence among these recently integrated sequences is 2.6% (1.5% at nucleotide level), while proviruses that integrated 20 or 30 million years ago can differ from these and from each other by 15%. Using only reads that map uniquely to one provirus (have a single ‘best’ match) reduces the apparent contribution of potentially Env-encoding proviruses around ten-fold (illustrated in Additional file 4). This potential artifact has been elegantly shown by simulations in Bhardwaj et al. [34]. Our laboratory and bioinformatic pipeline is illustrated in Fig. 2.

### Western blotting

We used a mouse anti-HML-2 Env mAB called HERM-1811-5 purchased from Austral Biologicals, San Ramon, CA, USA. This antibody has been used by several different groups in transfection experiments of HML-2 *env* with detection by Western blotting [40, 57–59], and it has also been used in FACS [60] and IHC staining [6, 7, 61]. Epitope mapping shows the antibody to bind to Env’s constituent Trans-Membrane (TM) sub-unit [7]. Western blotting in previous studies report the uncleaved Env full protein to be 70–95 kDa [23, 28, 57] and the TM sub-unit to be 26-43 kDa depending upon their glycosylation state [28, 57]. The strong ~ 55 kDa band is non-specific (unpublished data).

For HML-2 Env immunoblotting, we also used MCF7 cells purchased from ATCC (Manassas, VA, USA). Cells were lysed on ice using RIPA buffer (25 mM Tris-HCl pH 7.6, 150 mM NaCl, 1% NP-40, 1% sodium deoxycholate, 0.1% SDS) containing complete protease inhibitors (Sigma Aldrich, working stock made by dissolving 1 tablet in 2 ml of distilled water), and both phosphatase inhibitors Cocktails B & C (Santa Cruz). Lysed cells were centrifuged at 13000 rpm for 15 min to remove cellular debris. Protein concentrations were measured using BCA protein assay (Biorad). Thirty micrograms of proteins were separated by SDS–PAGE on a polyacrylamide gel in reducing buffer (4 x reducing buffer: 250 mM Tris–HCl pH 6.8, 8% SDS (Fisher Scientific), 40% glycerol (Sigma Aldrich), 200 mM DTT, bromophenol blue) and transferred onto a polyvinylidene difluoride (PVDF) membrane (BioRad). The membrane was blocked in Tris Buffer Saline, 0.1% Tween (Sigma Aldrich), 5% skimmed milk (Sigma Aldrich) and 2% Bovin Serum Albumin (Fisher Scientific). The membrane was incubated overnight with the HERM-1811-5 mAB (1:500) and incubated for 1 h the next day with anti-mouse secondary antibodies. ECL (Amersham) was used for detection.

### Alignment of HML-2 proviruses

Initially, sequences were taken from Subramanian et al. [20] and confirmed by manual comparison to the human reference sequence (hg38) using the UCSC Genome Browser (https://genome.ucsc.edu). We also added four sequences from more recently discovered proviruses, 8q24.3c, 19p12d, 19p12e and Xq21.33 [18] (kindly sent by the authors).

There are 28 full-length proviruses known from the main HML-2 clade that integrated in the last 5 million years, i.e. since the divergence from the chimpanzee (= LTR5-Hs clade [18, 20]; we ignore here a few proviruses in a second clade that are also human-specific but are old and were copied by segmental duplication). Of these 28 proviruses, five lack a complete *env* sequence and two (3q21.2 and 21q21.1) have been hypermutated prior to integration by one of our innate immune system proteins, APOBEC3G [62], and as a result have many premature stop codons (see Additional file 1) so we ignored them. Alignment of all these recently integrated sequences was unambiguous and done manually in MEGA versions 5 and 6 [63, 64]. We present the alignment of the remaining 21 *env* sequence in Additional file 9 and a NJ tree of them in Additional file 5. From this alignment, a single conserved HML-2 *env* sequence was constructed manually for the probe design. Eleven of these proviruses are type 1, which – as discussed above – are defined by having a 292 nucleotide deletion near the beginning of *env*, which takes the gene out of its correct reading frame. However, this deletion removes the *pol* stop codon and puts the *env* sequence downstream of the deletion back in frame with *pol*. Such resulting Pol-Env fusion proteins would be detected by long-read proteomic methods so we include the sequences here.

### Target library preparation and enrichment

Total cellular RNA was extracted using TRIzol reagent (Ambion, Life Technologies) according to the manufacturer’s guidelines. After isopropanol precipitation and washing with 75% ethanol, the RNA was further purified using the GeneJET RNA Purification Kit (Thermo Scientific). Poly(A) RNA was selected using the Dynabeads mRNA DIRECT Micro Kit (Life Technologies).

For the short-read sequencing, 200-300 ng of poly(A) RNA was fragmented with RNaseIII (Life Technologies) for 2 or 10 min (see Additional file 3) and fragment libraries were prepared from 50 to 100 ng fragmented RNA according to the Ion Total RNA-Seq Kit v2 protocol (Life Technologies). Prior to enrichment, library amplification was performed with the Ion 5′ Primer v2 and Ion 3′ Primer v2 with 45 μl Platinum® PCR SuperMix High Fidelity in a total volume of 53 μL. Amplification cycles were as follows: 94 °C for 2 min, [94 °C for 30 s, 50 °C for 30 s, 68 °C for 30 s] × 2 cycles, [94 °C for 30 s, 62 °C for 30 s, 68 °C for 30 s] × 16 cycles, 68 °C for 5 min. Agilent’s eArray was used to create enrichment baits. The HML-2 consensus sequence at 7536 nucleotides is relatively short so a tiling frequency of × 10 was used to give good coverage. Hybrid capture was performed with 130-160 ng of the fragment library, concentrated to 3.4 μl using a Speedvac (Eppendorf), and 2 μl of the SureSelect XT RNA bait library in 27 μL at 65 °C for 18-20 h according to the SureSelect Target Enrichment System Protocol (Agilent). After hybridisation, the enriched fragment library was captured using streptavidin beads (Dynabeads MyOne Streptavidin T1, Invitrogen) and purified, also according to the same enrichment protocol. The purified, enriched fraction was amplified on the streptavidin beads using Herculase II Fusion DNA Polymerase (Agilent) according to the manufacturer’s instructions and with the following cycles: 98 °C for 2 min, [98 °C for 30 s, 60 °C for 10 s, 72 °C for 1 min] × 12 cycles, 72 °C for 10 min. The amplified captured library was finally purified with Agencourt AMPure XP beads (Beckman Coulter), and quantified by real-time PCR for later sequencing.

For the long-read sequencing, 300 ng of mRNA was synthesised into double-stranded cDNA using the Roche cDNA synthesis kit according to the manufacturer’s instructions. 180 ng of double-stranded cDNA was end repaired using the Ion Plus Fragment Library Kit (Life Technologies) and size selected, to remove DNA below 1 kb, using Ampure XP beads. Ion PGM adapters were then ligated onto the DNA using the Ion Plus Fragment Library Kit. Library amplification was as above except that 50 μl PCR SuperMix was used in a total volume of 60 μL, with amplification cycles of 94 °C for 2 min, [94 °C for 20 s, 58 °C for 15 s, 70 °C for 10 min] × 30 cycles, 70 °C for 10 min. Hybrid capture was performed with 840 ng of the fragment library, and the purified, enriched fraction was amplified with the following cycles: 94 °C for 5 min, [94 °C for 20 s, 58 °C for 20 s, 70 °C for 10 min] × 30 cycles, 70 °C for 10 min. The amplified captured library was then quantified using the Qubit high sensitivity kit for sequencing on the MinION.

### Sequencing of enriched bait library

For short-read sequencing, 26pM of amplified library was submitted to emulsion PCR on the Ion OneTouch™ 2 instrument using the Life Technologies Ion PGM™ Template OT2 200 kit (or OT2 400 kit for 400 bp libraries) according to the manufacturer’s instructions. We note that reducing RNA digestion time from 10 min to 2 min only resulted in median read lengths increasing from 91 and 100 bp (G519 and JVM2 growth 2 respectively) to 121, 134 and 132 bp (JVM2 growth 1, JVM2 growth 3 and REC1 respectively). All JVM2 experiments were carried out with the 400 bp kit. Ion sphere particles (ISPs) were enriched using the ES instrument, then loaded and sequenced on an Ion 316v2 Chip (Life Technologies). The Run Summary files from the Ion Torrent are presented in Additional file 3. For long-read sequencing, 2 × 2.5 μg of amplified capture library of size range ~ 0.5 kb to 8 kb was prepared for 1d2 sequencing using the LSK308 sequencing kit. Briefly end repair and the first ligation were performed as per the standard protocol, with the exception that 0.4 volumes of AMPXL were used in each case to reduce the amount of DNA > 1.5kb recovered. Final libraries (12ul) at a concentration of 3.4 ng/μl and 0.5 ng/μl were combined with 35 μl RBF, 2.5 μl LBs and 2.5 μl water and loaded through the spot-on port into a pre-primed R9.5.1 flow cell. A second library was loaded at 16 h. Reads were acquired over 48 h using Min107 LSK308 48 h protocol with MinKnow Windows version 18.7.2. Albacore basecaller version 2.3.1(Ubuntu 16.04.4) was used to call 1d2 and 1d reads.

### Bioinformatic pipeline

After sequencing, short-read Quality Control (QC) was performed using the CLC Genomics Workbench software with low quality (Quality score < 0.05) and abnormally long (> 200 bp or > 400 bp depending on the sequencing kit used) or abnormally short reads (< 50 bp) excluded from each dataset. As mentioned in the Overview, reads were mapped to HML-2 proviruses in a ‘faux’ genome consisting only of concatenated HML-2 sequences using Bowtie2 [55], run within Tophat2 [65], and counted using Cufflinks [56] (all with default settings) and reporting the RPKM values (to take into account variation in provirus length and total number of reads). Almost identical results for the 87 proviruses in the reference genome sequence were obtained using HML-2 coordinates in the hg19 assembly rather than building a faux genome (not shown). Counting mappings to proviruses using featureCounts [66] rather than Cufflinks gave similar results (Additional file 10). Here, featureCounts is run with its default setting of only counting uniquely mapping reads, so it underestimates the expression of more similar proviruses (we obtain the same results with Cufflinks if multi-mapping reads are removed prior to analysis using Samtools [67]). The multi-mapping option in featureCounts (−M) was not used because it allocates each multi-mapping read to all possible proviruses, so leading to their overestimation (e.g. if one read maps equally well to 10 proviruses, it is counted 10 times). Long reads in FASTQ format were all mapped to the same faux genome as above using minimap2 [68] (QC was applied later only for mapping to unique SNP alleles – see below).

We also used another method to quantify the relative expression of proviruses, based on k-mers pseudoalignments, implemented in the software package kallisto [69]. The reference sequences were transformed into indexes with k-mer length 31 and quantification was done with the default parameters. The reads' abundance values in TPMs produced by the program were then further normalized with variance-stabilizing transformation (DESeq2 Bioconductor package). This approach gave broadly similar results to those presented in Figs. 3a and 4 but, similar to using featureCounts in default mode, it also appears to underestimate the contribution of more similar proviruses (Additional file 10).

### Comparison of mapping results with searches for matches to unique SNPs

Bowtie2 maps short reads either *uniquely* (= there is a single best match) or to more than one provirus (the so-called *multi-mappers* that map equally well to more than one provirus). By default, Bowtie2 randomly allocates multi-mappers. The only other method of allocating multi-mapping reads to potential proviruses is to do so proportional to the number of uniquely mapping reads that each provirus has. However, this alternate method would be misleading for endogenous retroviruses (and other transposable elements) because it would be biased towards older proviruses, which by definition would have had more time in which to accrue mutations and hence more uniquely mapping reads. When run with default parameter values, only a minority of uniquely or multi-mapping reads match their best provirus *perfectly*, i.e. their alignment requires no insertions of gaps or nucleotide substitutions). We counted the number of uniquely mapping reads using featureCounts (in default mode) for each Env-encoding provirus (Table 3). We then searched for unique SNPs in the *env* sequences (only) as follows and added them to this table: firstly, we selected all unique SNPs (single nucleotide polymorphisms) from the alignment in Additional file 9; secondly, we checked that these were unique among all known HML-2 sequences by searching in a FASTA file of these sequences with a 31 nt long sequence that spanned the SNP (15nts either side) using a grepping procedure carried out in a Python script; finally, exact matches to each of these 31 nt sequences in our FASTQ files were then found and counted using the above grepping procedure (we ignored REC1 because of the small number of mapping reads). In a few instances, e.g. T(1677)C in 5p13.3, the coordinate represents one of two SNPs within the 31 nt sequence, the combination of which is unique.

We repeated the above analysis for long reads except for excluding poorly mapping reads (Q = 20) in featureCounts and in the SNP counting we used a 17 nt rather than a 31 nt long sequence. Both of these adjustments were necessary because of the much higher error rate with long-read sequencing. We also wrote a python script to detect long reads that contained multiple unique SNP alleles (in this case using an 11 nt long sequence). Allowing for mismatches in the regions flanking the SNP did not provide additional insights.

## Supplementary information

**Additional file 1:** Figure showing interruptions of ORFs among HML-2 proviruses in the human reference genome that integrated since the human-chimpanzee divergence.

**Additional file 2:** Comparison of our results to those from whole transcriptome datasets in the Short Read Archive (SRA).

**Additional file 3:** Ion Torrent run summaries.

**Additional file 4:** Figure illustrating problem in mapping reads to proviruses that have similar sequences.

**Additional file 5:** Phylogenetic tree of all HML-2 proviruses that have complete *env* sequences and that integrated since the human-chimpanzee divergence (~ 5 million years ago).

**Additional file 6:** Further details of other published RNA expression studies and further discussion of ours.

**Additional file 7:** Guidance on future proteomic work.

**Additional file 8:** Alignment used to build probe sequence (= ‘Consensus’).

**Additional file 9:** Alignment of *env* gene from HML-2 proviruses in FASTA format. Gaps inserted to maintain reading frame (first 3 nucleotides are methionine).

**Additional file 10:** Comparison of results of three different methods for measuring the relative provirus abundance in the JVM2_growth2 FASTQ file.

**Additional file 11:** Comma delineated text file (.csv) of raw data matrix used to generate the pie charts shown in Fig. 4.

## Data Availability

The FASTQ datasets generated during this study are available in the NCBI’s SRA (Short Read Archive) repository under BioProject ID PRJNA357368 (accession numbers SRR5109951 to SRR5109954 inclusive).

## Abbreviations

FACS: Fluorescence Activated Cell Sorting
GREP: Globally search a Regular Expression and Print
HML-2: Human Endogenous Retrovirus type K(HML-2)
IHC: ImmunoHistoChemistry
LTR: Long Terminal Repeat
mAB: monoclonal antibody
MaLR: Mammalian apparent LTR Retrotransposon
MCL: Mantle Cell Lymphoma
ORF: Open Reading Frame
RPKM: Reads Per Kilobase of transcript per Million mapped reads
SAAV: Single Amino Acid Variant
SIV: Simian Immunodeficiency Virus
SMRT: Single Molecule Real Time
SNP: Single Nucleotide Polymorphism
SRA: Short Read Archive
TAA: Tumour-Associated Antigen
UCSC: University of California Santa Cruz
