# Correction to: Characterising a human endogenous retrovirus(HERV)-derived tumour-associated antigen: enriched RNA-Seq analysis of HERV-K(HML-2) in mantle cell lymphoma cell lines

**DOI:** 10.1186/s13100-020-00226-8

**Published:** 2020-11-30

**Authors:** Witold Tatkiewicz, James Dickie, Franchesca Bedford, Alexander Jones, Mark Atkin, Michele Kiernan, Emmanuel Atangana Maze, Bora Agit, Garry Farnham, Alexander Kanapin, Robert Belshaw

**Affiliations:** 1 Peninsula Medical School, Faculty of Health: Medicine, Dentistry and Human Sciences, University of Plymouth, Plymouth, UK; 2 School of Biomedical Sciences, Faculty of Health: Medicine, Dentistry and Human Sciences, University of Plymouth, Plymouth, UK; 3 Department of Oncology, University of Oxford, Oxford, UK; 4 Current address: Institute of Translational Biomedicine, Saint Petersburg State University, Saint Petersburg, Russia

**Correction to: Mobile DNA 11, 9 (2020)**

**https://doi.org/10.1186/s13100-020-0204-1**

Following publication of the original article [1], the authors requested to add the below sentence in the “Funding” section.

Alexander Kanapin (AK) was supported by funding for the project #51148284 from Saint Petersburg State University, Saint Petersburg, Russia.
