# Supplementary material for: Characterising a human endogenous retrovirus(HERV)-derived tumour-associated antigen: enriched RNA-Seq analysis of HERV-K(HML-2) in mantle cell lymphoma cell lines
Source: Mob DNA. 2020 Feb 7;11:9. doi: 10.1186/s13100-020-0204-1 (PMC7007669; doi:10.1186/s13100-020-0204-1)
Supplement: Supplementary file 1 — Additional file 1: Figure showing interruptions of ORFs among HML-2 proviruses in the human reference genome that integrated since the human-chimpanzee divergence. [file 13100_2020_204_MOESM1_ESM.docx]

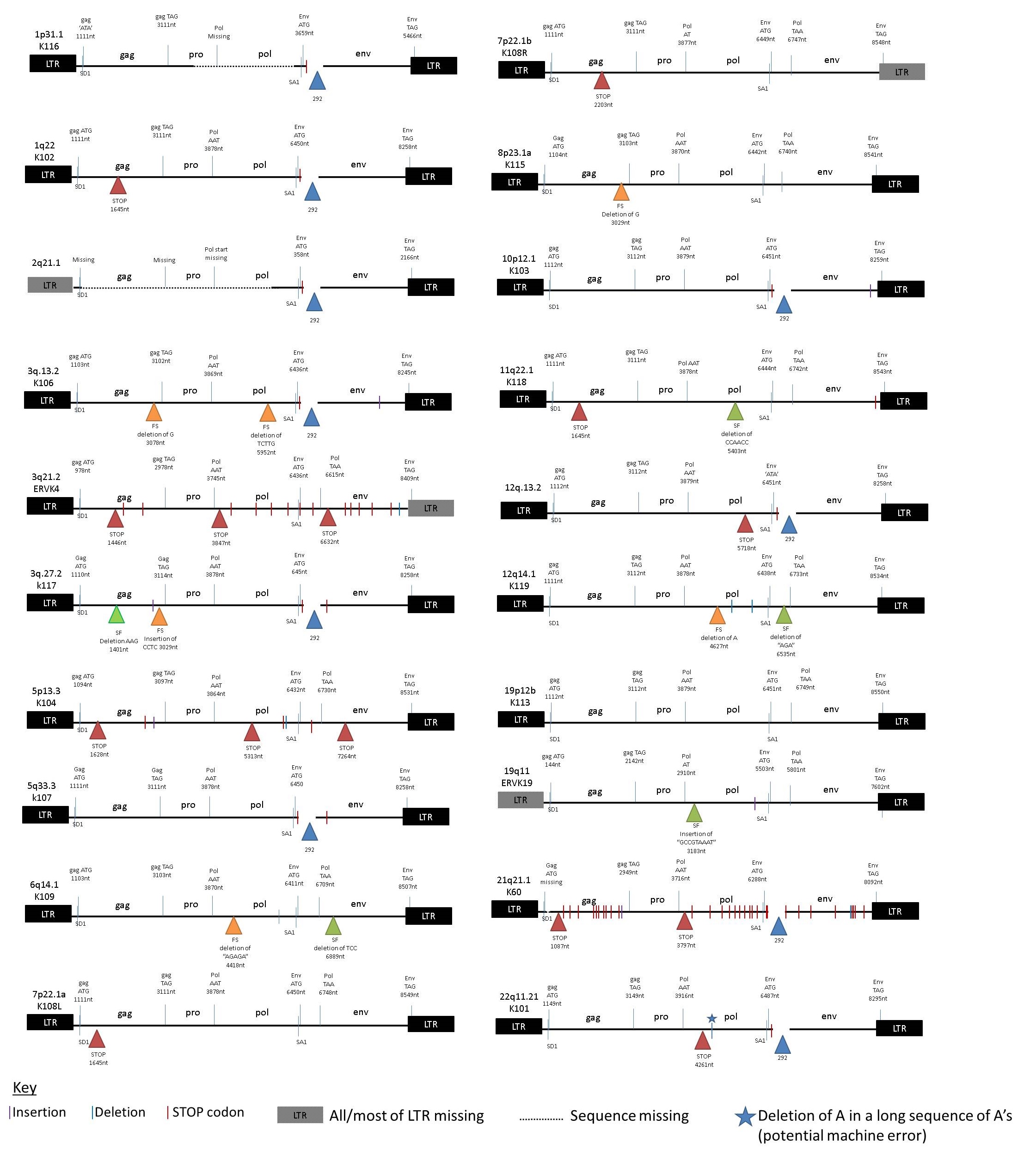


**Figure showing interruptions of ORFs among HERV-K(HML-2) proviruses in human reference genome sequence that integrated since the human-chimpanzee divergence, ~5 million years ago**. Key mutations are shown as triangles: dark red show premature stop codons (where there are more than one in a gene the subsequent ones are shown by vertical dark red lines); orange and green triangles show indels (insertion/deletions) that shift or maintain the frame respectively, and blue shows the 292 nt deletion that defines Type 1 proviruses. The smaller blue star is a deletion among a long A homopolymer so is probably a sequencing error, as may be the frame-shift deletions in 3q.27.2 and 8p23.1a. Dotted lines and grey boxes both show missing sequences.
