# Supplementary material for: Characterising a human endogenous retrovirus(HERV)-derived tumour-associated antigen: enriched RNA-Seq analysis of HERV-K(HML-2) in mantle cell lymphoma cell lines
Source: Mob DNA. 2020 Feb 7;11:9. doi: 10.1186/s13100-020-0204-1 (PMC7007669; doi:10.1186/s13100-020-0204-1)
Supplement: Supplementary file 2 — Additional file 2: Comparison of our results to those from whole transcriptome datasets in the Short Read Archive (SRA). [file 13100_2020_204_MOESM2_ESM.docx]

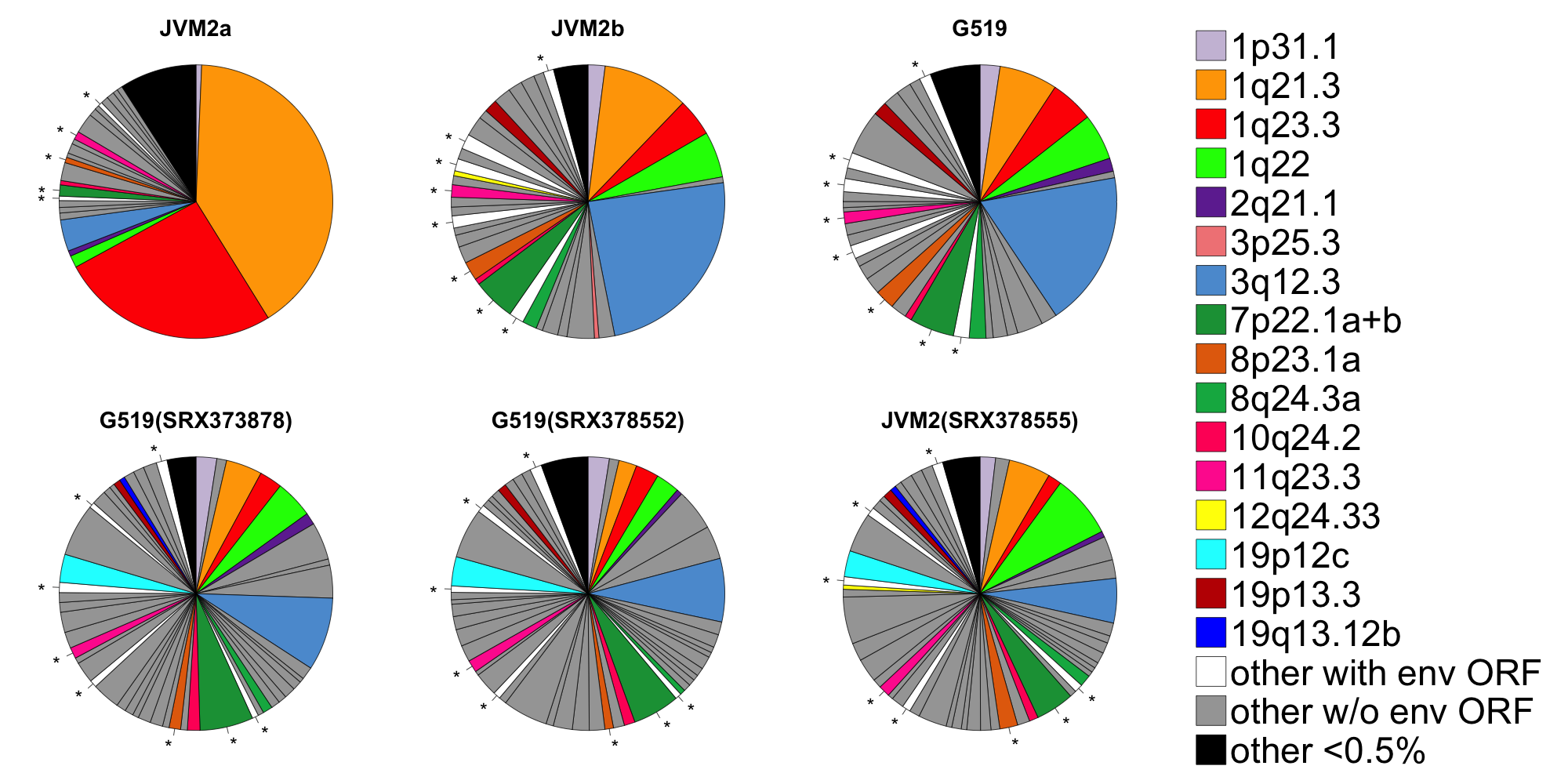


**Figure comparing results to those from whole transcriptome datasets in the Short Read Archive (SRA).** As shown above (top line; same as in figure 4), there is a strong effect of experiment on the pattern of our estimated provirus expression. Results from one analysis of JVM2 (labeled b) are more similar to the G519 sequenced at the same time and run on the same chip than to the results of the same line (labeled a) grown and analysed at a different time, although under an almost identical protocol (see Methods).

The similarity between one JVM2 run and the G519 are not unique. We found two publicly available whole transcriptome RNA-Seq studies of our cell lines at the NCBI's SRA (Short Read Archive; https://www.ncbi.nlm.nih.gov/sra): GEO BioProjects PRJNA181354 (Novartis Institutes for BioMedical Research; no publication) and PRJNA227025 (= Series GSE52148) [1]. We carried out our normal QC and Bowtie2 mapping on these Illumina HiSeq FASTQ datasets and find that the overall expression patterns from G519 and JVM2 in the same experiment (SRX378552 and SRX378555 from PRJNA181354) are broadly as similar to each other as the G519 is to the same cell line analysed by a different group (SRX373878 from PRJNA227025).

We consider these whole transcriptome SRA datasets to be less reliable than ours. Firstly, there appears to be a major artifact: the old provirus 9q34.3 (also found in the Orangutan) accounted for 76-80% of the reads assigned to HERV-K(HML-2) in these three datasets, and 67% in our unenriched JVM2 one. In contrast, this provirus has less than 0.1% of the reads assigned to it in our enriched analyses. We suspect that this is an artifact caused by the provirus containing an Alu integration [2], with many reads from Alu transcripts being erroneously mapped to it. Our use of a probe designed from the canonical HERV-K(HML-2) proviral sequence appears to avoid this. Secondly, after deleting the hits to this single provirus for our analysis shown in the above figure, we were left with a total of only 1,500-8,000 reads assigned to HERV-K(HML-2) in each SRA dataset.

To confirm that similar artefacts were not affecting our enriched data, we compared the sequences of our highly expressed proviruses (1q21.3, 1q23.3 and 3q12.3) to a well-studied reference, 19p12b (K113), using BLAST and found that they contained no insertions longer than a few nucleotides.

1. Kuo PY, Leshchenko VV, Fazzari MJ, Perumal D, Gellen T, He T, Iqbal J, Baumgartner-Wennerholm S, Nygren L, Zhang F *et al*: High-resolution chromatin immunoprecipitation (ChIP) sequencing reveals novel binding targets and prognostic role for SOX11 in mantle cell lymphoma. *Oncogene* 2015, **34**(10):1231-1240.

2. Subramanian RP, Wildschutte JH, Russo C, Coffin JM: Identification, characterization, and comparative genomic distribution of the HERV-K (HML-2) group of human endogenous retroviruse**s**. *Retrovirology* 2011, **8**:90.
