# Supplementary material for: Characterising a human endogenous retrovirus(HERV)-derived tumour-associated antigen: enriched RNA-Seq analysis of HERV-K(HML-2) in mantle cell lymphoma cell lines
Source: Mob DNA. 2020 Feb 7;11:9. doi: 10.1186/s13100-020-0204-1 (PMC7007669; doi:10.1186/s13100-020-0204-1)
Supplement: Supplementary file 3 — Additional file 3: Ion Torrent run summaries. [file 13100_2020_204_MOESM3_ESM.docx]

**
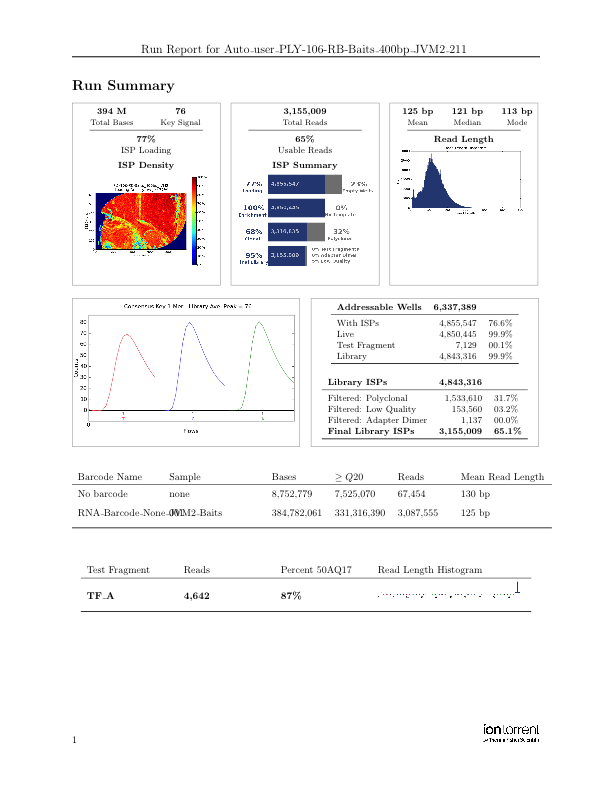
**

**Above: JVM2 growth 1 (2 mins digestion) run summary.**

**
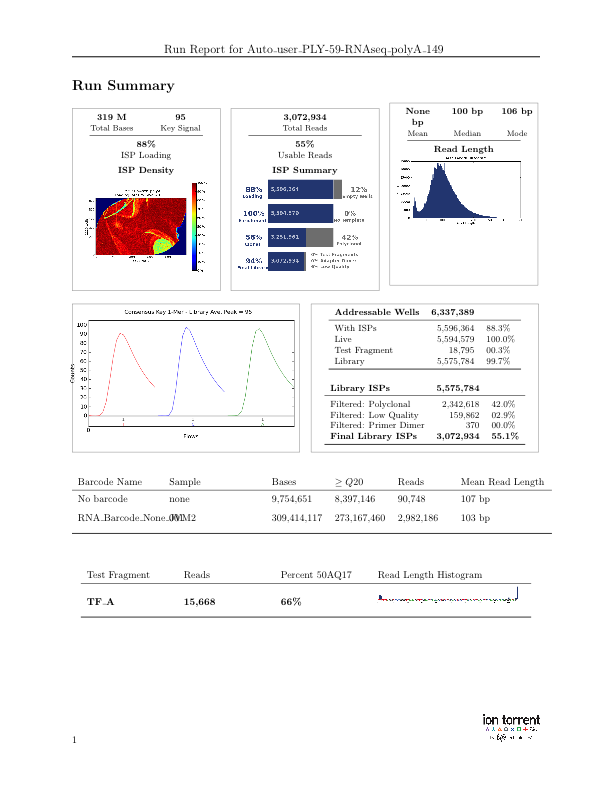
**

**Above: JVM2 growth 2 (10 mins digestion) run summary.**

**
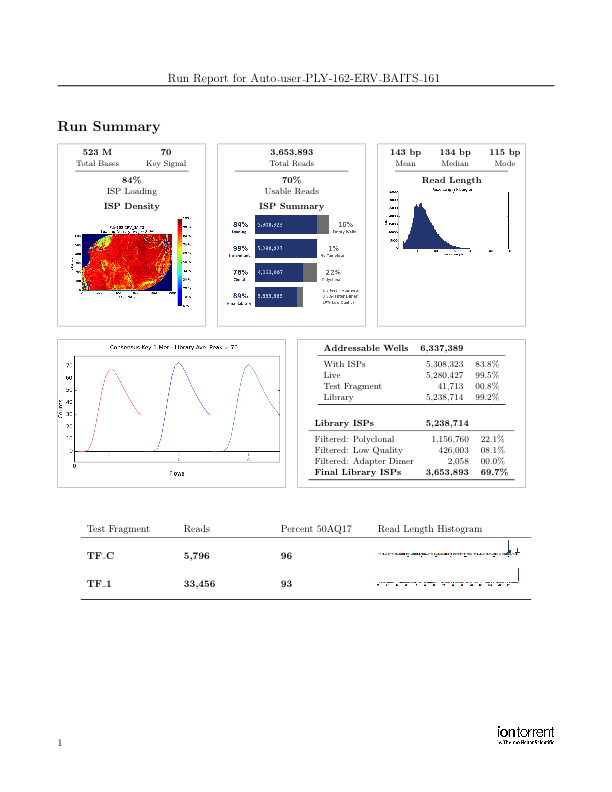
**

**Above: JVM2 growth 3 (2 minutes digestion) run summary.**

**
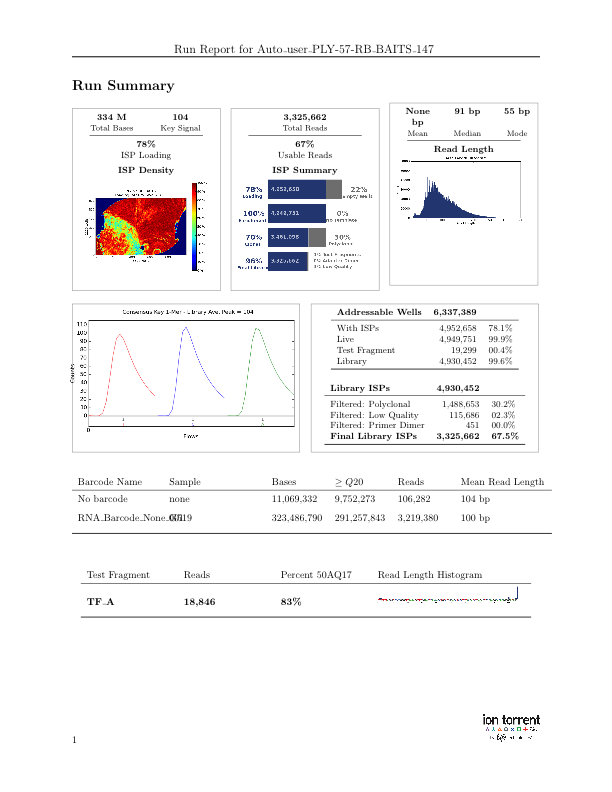
**

**Above: G519 (10 minutes digestion) run summary.**

**
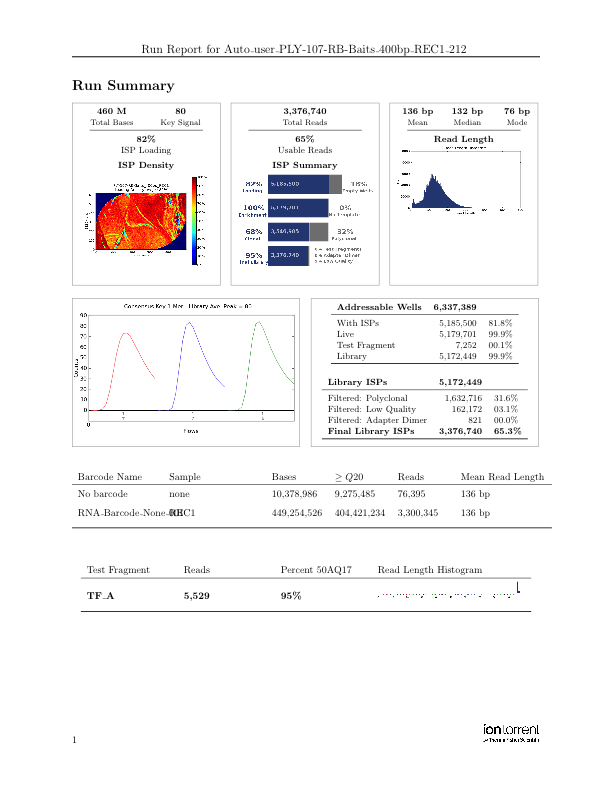
**

**Above: REC1 (2 mins digestion) run summary.**
