# Supplementary material for: Characterising a human endogenous retrovirus(HERV)-derived tumour-associated antigen: enriched RNA-Seq analysis of HERV-K(HML-2) in mantle cell lymphoma cell lines
Source: Mob DNA. 2020 Feb 7;11:9. doi: 10.1186/s13100-020-0204-1 (PMC7007669; doi:10.1186/s13100-020-0204-1)
Supplement: Supplementary file 4 — Additional file 4: Figure illustrating problem in mapping reads to proviruses that have similar sequences. [file 13100_2020_204_MOESM4_ESM.docx]

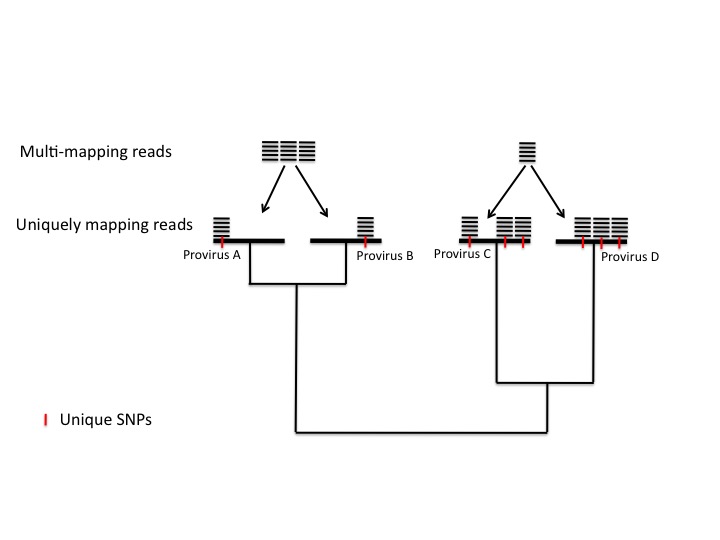


**Illustration of problem found in mapping reads to proviruses that have similar sequences.** Proviruses A and B are closely related (diverged from a common ancestor recently) and so have few unique SNPs. Few reads therefore map uniquely to either provirus A or B but many reads map equally well to both. In contrast, proviruses C and D are more distantly related to each other and so have many unique SNPs. Many reads therefore map uniquely to either provirus C or D but few reads map equally well to both.
