# Supplementary material for: Characterising a human endogenous retrovirus(HERV)-derived tumour-associated antigen: enriched RNA-Seq analysis of HERV-K(HML-2) in mantle cell lymphoma cell lines
Source: Mob DNA. 2020 Feb 7;11:9. doi: 10.1186/s13100-020-0204-1 (PMC7007669; doi:10.1186/s13100-020-0204-1)
Supplement: Supplementary file 5 — Additional file 5: Phylogenetic tree of all HML-2 proviruses that have complete env sequences and that integrated since the human-chimpanzee divergence (~ 5 million years ago). [file 13100_2020_204_MOESM5_ESM.docx]

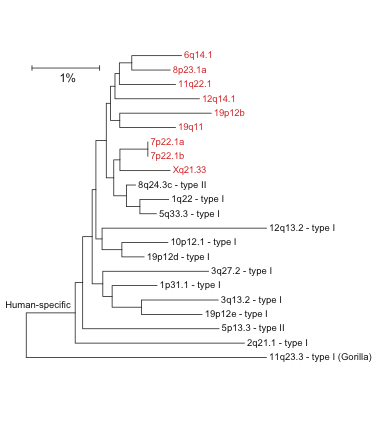


**Phylogenetic tree of all HERV-K(HML-2) proviruses that have complete *env* sequences and that integrated since the human-chimpanzee divergence (~ 5 million years ago).** Tree is built from *env* amino acid sequences using the Neighbor-Joining method (as implemented in MEGA5) and midpoint rooted with one additional provirus that is also found in the Gorilla genome. Proviruses with full-length *env* ORFs (see table 1) are shown in red. Scale shows amino acid divergence along branches.
