# Supplementary material for: Characterising a human endogenous retrovirus(HERV)-derived tumour-associated antigen: enriched RNA-Seq analysis of HERV-K(HML-2) in mantle cell lymphoma cell lines
Source: Mob DNA. 2020 Feb 7;11:9. doi: 10.1186/s13100-020-0204-1 (PMC7007669; doi:10.1186/s13100-020-0204-1)
Supplement: Supplementary file 6 — Additional file 6: Further details of other published RNA expression studies and further discussion of ours. [file 13100_2020_204_MOESM6_ESM.docx]

**Further details of other RNA expression studies**

Figure 4 in the main text compares the relative expression of individual HERV-K(HML-2) proviruses with values taken from a range of other studies (cited in the main text). With one exception, these studies used methods that all involve an initial RT-PCR of a particular HERV-K(HML-2) region: (a) Sanger Sequencing following cloning plus direct NGS of HERV-K(HML-2)-specific amplified cDNA from prostate tumours [1], (b) SMRT (Single Molecule Real-Time) sequencing of HERV-K(HML-2)-specific amplified cDNA from peripheral blood mononuclear cells (PBMCs) of healthy donors [2], (c) Sanger Sequencing following cloning of HERV-K(HML-2)-specific amplified cDNA from melanoma and associated lymph node metastases [3] – here we also pooled results from several other tumor types published in a later study by the same group [4].

Firstly, there are a few nomenclatural problems in identifying proviruses across studies. Converting the hg18 coordinates in Schmitt et al. [3] to hg19 allowed us to identify and use the Subramanian et al. [5] nomenclature for results in that study: of note, ERVK-14 is 7q22.2, ERVK-26 is 8p23.1d; ERVK-28 is 19q11 (although reported as ERVK-19 in [5] it has the full-length *gag* and *env* ORFs reported in the Schmitt et al. study); ERVK29 is 19q13.12b (as is the ERVK29 of [1]). Because of sequence similarity in the amplified region, HERV-K27 and HML-2_8p23.1 (our 8p23.1b and 8p23.1c respectively) were not distinguished in the results of Goering at el. [1], so we distributed the (few) reads equally between the two proviruses.

Comparisons between tissues are made harder by differences in the experimental method used. As mentioned in the main text, a provirus we find to be highly expressed is missed in the healthy lymphocyte study because it lacks the primer binding sites. Also, comparing the results of SMRT and Sanger Sequencing of cloned cDNA transcripts on the same Prostate cell lines shows very large differences (fig. 2 in the Goering et al. study [1]). This is due to different primer sequences being used: SMRT follows RT-PCR using primers matching the common provirus 22q11.23 (dark blue in figure 4, also called provirus "H22q") while the Sanger sequencing follows RT-PCR using primers from Schmitt et al. [3] that have 4-8 mismatches with this provirus [1]. The high expression of 22q11.23 in prostate and testes cancer is driven almost entirely by an upstream second HER-V provirus creating an identified lincRNA (Long Intervening NonCoding RNA) [6] – which it could be argued is therefore not a lincRNA [1]. Similarly, the teratocarcinoma RNA-Seq dataset shows high expression of the proviruses 19p12c and 22q11.23, but these do not appear in the seminoma in figure 4 because they were not scored in that study.

The relatively high expression of 1q32.2 in our Rec1 is puzzling because this provirus lacks the 5' LTR, which contains the promoter motifs. In theory, high expression might result from it by being within the last intron of the CR1 gene or an unidentified long non-coding RNAs derived from the multiple nearby Alu elements (the very close LTR belonging to another ERV lineage – 913bp upstream – is in reverse orientation). More detailed analysis of the reads could resolve this question. However, we note that a much smaller proportion of reads were assigned to HERV-K(HML-2) than in the other cell lines and this provirus is not highly expressed (<5% of RPKM) in another Rec1 dataset from the SRA (SRX378549; unpublished; not shown).

**References**

1. Goering W, Schmitt K, Dostert M, Schaal H, Deenen R, Mayer J, Schulz WA: Human endogenous retrovirus HERV-K(HML-2) activity in prostate cancer is dominated by a few loci. *Prostate* 2015, 75:1958-1971.

2. Brinzevich D, Young GR, Sebra R, Ayllon J, Maio SM, Deikus G, Chen BK, Fernandez-Sesma A, Simon V, Mulder LCF: HIV-1 interacts with Human Endogenous Retrovirus K (HML-2) Envelopes derived from human primary lymphocytes. *J Virol* 2014, 88:6213-6223.

3. Schmitt K, Reichrath J, Roesch A, Meese E, Mayer J: Transcriptional profiling of Human Endogenous Retrovirus group HERV-K(HML-2) loci in melanoma. *Genome Biol Evol* 2013, 5:307-328.

4. Flockerzi A, Ruggieri A, Frank O, Sauter M, Maldener E, Kopper B, Wullich B, Seifarth W, Müller-Lantzsch N, Leib-Mösch C *et al*: Expression patterns of transcribed human endogenous retrovirus HERV-K(HML-2) loci in human tissues and the need for a HERV Transcriptome Project. *BMC Genomics* 2008, 9:354.

5. Subramanian RP, Wildschutte JH, Russo C, Coffin JM: Identification, characterization, and comparative genomic distribution of the HERV-K (HML-2) group of human endogenous retroviruses. *Retrovirology* 2011, 8:90.

6. Bhardwaj N, Montesion M, Roy F, Coffin JM: Differential expression of HERV-K (HML-2) proviruses in cells and virions of the Teratocarcinoma cell line Tera-1. *Viruses* 2015, 7:939-968.
