# Supplementary material for: Characterising a human endogenous retrovirus(HERV)-derived tumour-associated antigen: enriched RNA-Seq analysis of HERV-K(HML-2) in mantle cell lymphoma cell lines
Source: Mob DNA. 2020 Feb 7;11:9. doi: 10.1186/s13100-020-0204-1 (PMC7007669; doi:10.1186/s13100-020-0204-1)
Supplement: Supplementary file 7 — Additional file 7: Guidance on future proteomic work. [file 13100_2020_204_MOESM7_ESM.docx]

**Guidance on future proteomic work**

The standard so-called 'bottom-up', proteomic methods will be unsuitable for this purpose because they generate short peptide sequences, identifying and counting single amino acid variants (SAAVs) from a purpose-built reference library, e.g. Nie et al. 2014 [1]. Such an analysis would leave us with the same problem we encountered here with short RNA reads. Even if we combine short read proteomics with sequencing of all the proviruses present in the tested individuals or cell lines, which is feasible, we would still require an algorithm to estimate provirus expression based on an observed frequency of single amino acid variants. Newer, so-called 'top-down', proteomic analysis methods do not require the protein to be cleaved [2] and could in theory return long Env amino acid sequences, which would then allow the generating provirus to be identified. Each of the known proviruses likely to be expressed at the protein level (shown in table 1) differ from each other by at least four amino acid substitutions in the 698 amino acid long Env protein (except for the identical tandem repeat provirus 7p22.1a+b). We suggest from our analyses here that in JVM2 we will recover no more than five proteoforms in an initial small study. The next step would be to allocate these to the translated amino acid sequences of the potentially *env*-encoding proviruses (alignment in Additional file 9). We might also encounter, and will need to distinguish, proteoforms resulting from the fused *pol* and *env* genes of Type 1 proviruses discussed above (which we assume would not contribute to the surface antigen). We therefore include these Type 1 *env* sequences in the alignment to help with this. Difficulties in allocation may result from the following four processes (illustrated in figure 5).

1. *Insertional polymorphism.* Proteoforms may come from proviruses that are represented in known genome sequences by the pre-integration site. Population screens suggest such novel proviruses will be rare [3].
2. *Solo LTR – full-length provirus polymorphism*. We are more likely to encounter proviruses with full-length *env* ORFs that are represented by solo LTRs in the reference genome. Solo LTRs are relict sequences lacking the internal protein-coding regions and result from a common within-provirus recombination event. They outnumber full-length proviruses (the typical retroviral 'provirus' form) in HERV-K(HML-2) by more than six to one – considering here only the more recently integrated (= human-specific) proviruses [4]. For example, provirus 10p12.1 exists as a solo LTR in the reference genome but the full-length proviral sequence is in GenBank [5].
3. *Gain of a full-length ORF.* Proteoforms may come from proviruses that lack full-length *env* ORFs in the sequenced genomes but – due to polymorphism at a premature stop codon or frameshift indel site – have them within the patient/cell line. Only two of the recently integrated proviruses have their *env* ORF interrupted by a single premature stop codon (5p13.3 and 8q24.3c in Additional file 5), and such gain of ORF events should be readily identifiable.
4. *Past recombination.* This will lead to novel chimaeric proteoforms containing single amino acid variants that previously appeared to be unique to different known proviruses. Previous work [6] suggested that the frequency of past recombination was low but our data suggest we will find multiple recombinants.

Any proteoforms that cannot be allocated to known proviruses will require further genomic analysis, and we suggest the following steps. Firstly, PCR the candidate full-length proviruses (i.e. non-solo LTR) in table 1, plus 5p13.3 and 8q24.3c, using primers matching their two genomic flanking sequences [7] and then sequence their *env* gene (using NGS or cloning followed by Sanger sequencing). If this fails to identify the origin of the proteoform, use a similar PCR screen to see if any recently integrated proviruses that are represented by solo LTRs in the human genome [7] are present as full-length proviruses in the patient/cell line. Such proviruses will appear as much longer fragments (~7kb rather than ~1kb) and the *env* can then be sequenced. Failure to identify a proteoform to a provirus after these steps suggests that a novel integration is present and its detection will require enriched whole genome sequencing similar to that used to detect novel LINEs (Long Interspersed Nuclear Elements) [8], another type of transposable element. An enriched genome long-read sequencing that both recovered flanking regions and allowed reconstruction of the full, ~10kb, HERV-K(HML-2) provirus could in theory both detect and characterise a novel provirus in one step. Further transcriptomic study would be unnecessary, although in the meantime higher accuracy in long-read technology would improve the type of analysis carried out in this study (we could also seek to reduce the number of rounds of PCR used to produce sufficient template because this can generate chimaeric reads as well as substitutions and indels).

**References**

1. Nie S, Yin HD, Tan ZJ, Anderson MA, Ruffin MT, Simeone DM, Lubman DM: Quantitative Analysis of Single Amino Acid Variant Peptides Associated with Pancreatic Cancer in Serum by an Isobaric Labeling Quantitative Method. J Proteome Res 2014, 13:6058-6066.

2. Tran JC, Zamdborg L, Ahlf DR, Lee JE, Catherman AD, Durbin KR, Tipton JD, Vellaichamy A, Kellie JF, Li M *et al*: Mapping intact protein isoforms in discovery mode using top-down proteomics. Nature 2011, 480:254-U141.

3. Wildschutte JH, Williams ZH, Montesion M, Subramanian RP, Kidd JM, Coffin JM: Discovery of unfixed endogenous retrovirus insertions in diverse human populations. Proc Natl Acad Sci USA 2016, 113:E2326-E2334.

4. Belshaw R, Watson J, Katzourakis A, Howe A, Woolven-Allen J, Burt A, Tristem M: Rate of recombinational deletion among human endogenous retroviruses. J Virol 2007, 81:9437-9442.

5. Subramanian RP, Wildschutte JH, Russo C, Coffin JM: Identification, characterization, and comparative genomic distribution of the HERV-K (HML-2) group of human endogenous retroviruses. Retrovirology 2011, 8:90.

6. Belshaw R, Pereira V, Katzourakis A, Talbot G, Paces J, Burt A, Tristem M: Long-term reinfection of the human genome by endogenous retroviruses. Proc Natl Acad Sci USA 2004, 101:4894-4899.

7. Belshaw R, Dawson ALA, Woolven-Allen J, Redding J, Burt A, Tristem M: Genomewide screening reveals high levels of insertional polymorphism in the human endogenous retrovirus family HERV-K(HML2): Implications for present-day activity. J Virol 2005, 79:12507-12514.

8. Shukla R, Upton KR, Munoz-Lopez M, Gerhardt DJ, Fisher ME, Thu N, Brennan PM, Baillie JK, Collino A, Ghisletti S *et al*: Endogenous retrotransposition activates oncogenic pathways in hepatocellular carcinoma. Cell 2014, 153:101-111.
