# Supplementary material for: Characterising a human endogenous retrovirus(HERV)-derived tumour-associated antigen: enriched RNA-Seq analysis of HERV-K(HML-2) in mantle cell lymphoma cell lines
Source: Mob DNA. 2020 Feb 7;11:9. doi: 10.1186/s13100-020-0204-1 (PMC7007669; doi:10.1186/s13100-020-0204-1)
Supplement: Supplementary file 10 — Additional file 10: Comparison of results of three different methods for measuring the relative provirus abundance in the JVM2_growth2 FASTQ file. [file 13100_2020_204_MOESM10_ESM.docx]

**
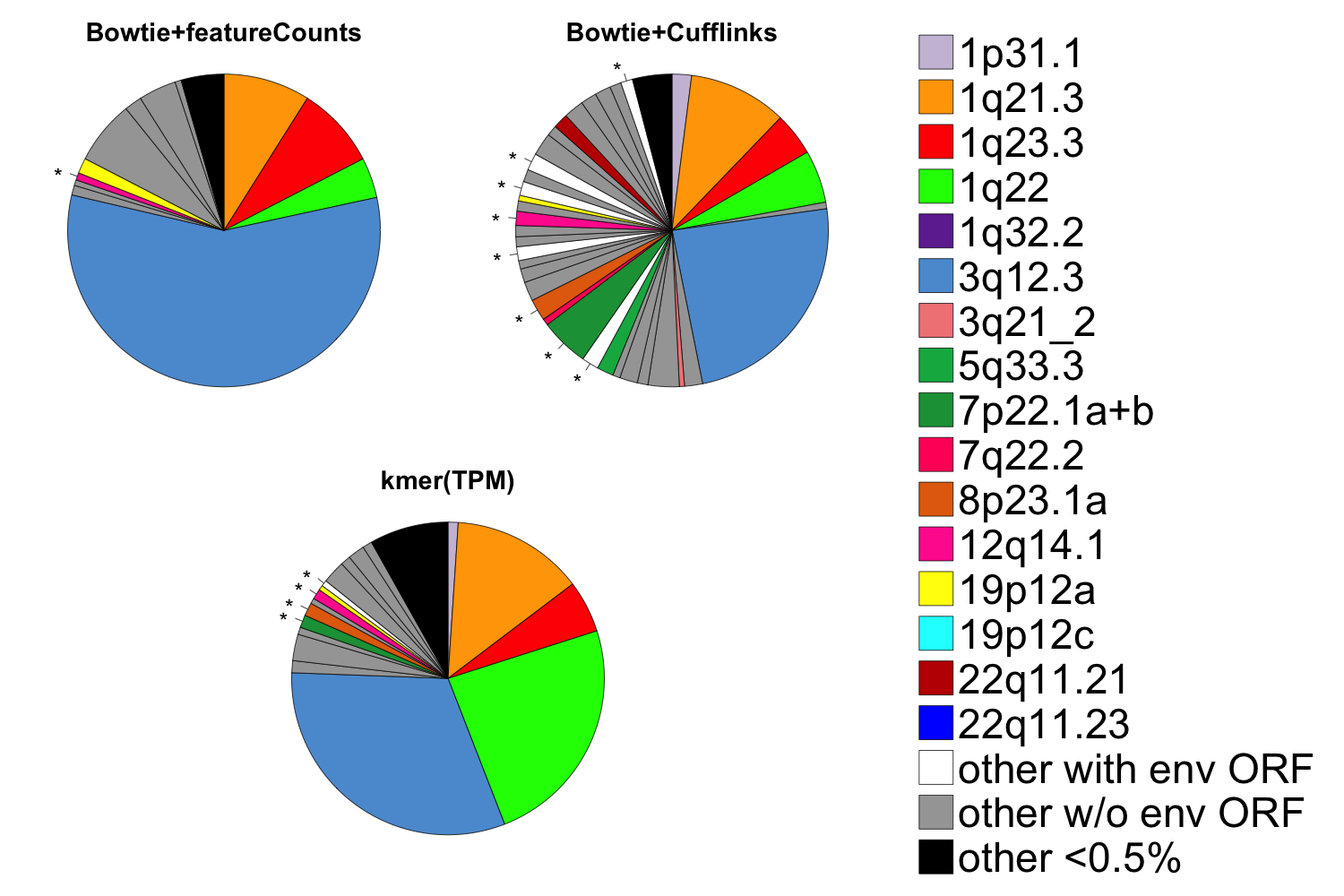
**

**Figure comparing results of three different methods for measuring relative provirus abundance in the JVM2b FASTQ file**. Reads were mapped to hg19 rather than the 'faux' genome used in the main analyses (eight proviruses are therefore not reported as they are not in the human genome reference sequence). The program featureCounts was run with the default option of only counting uniquely mapping reads (using the multiread mapping option, -M). This results in an overestimation of the expression of proviruses with more similar DNA sequences because each multimapping read is counted n times, where n is the number of possible equally good mappings.
